# Supplementary figures and images for: Sex-regulated gene dosage effect of PPARα on synaptic plasticity
Source: Life Sci Alliance. 2019 Mar 20;2(2):e201800262. doi: 10.26508/lsa.201800262 (PMC6427998; doi:10.26508/lsa.201800262)

Figure 1

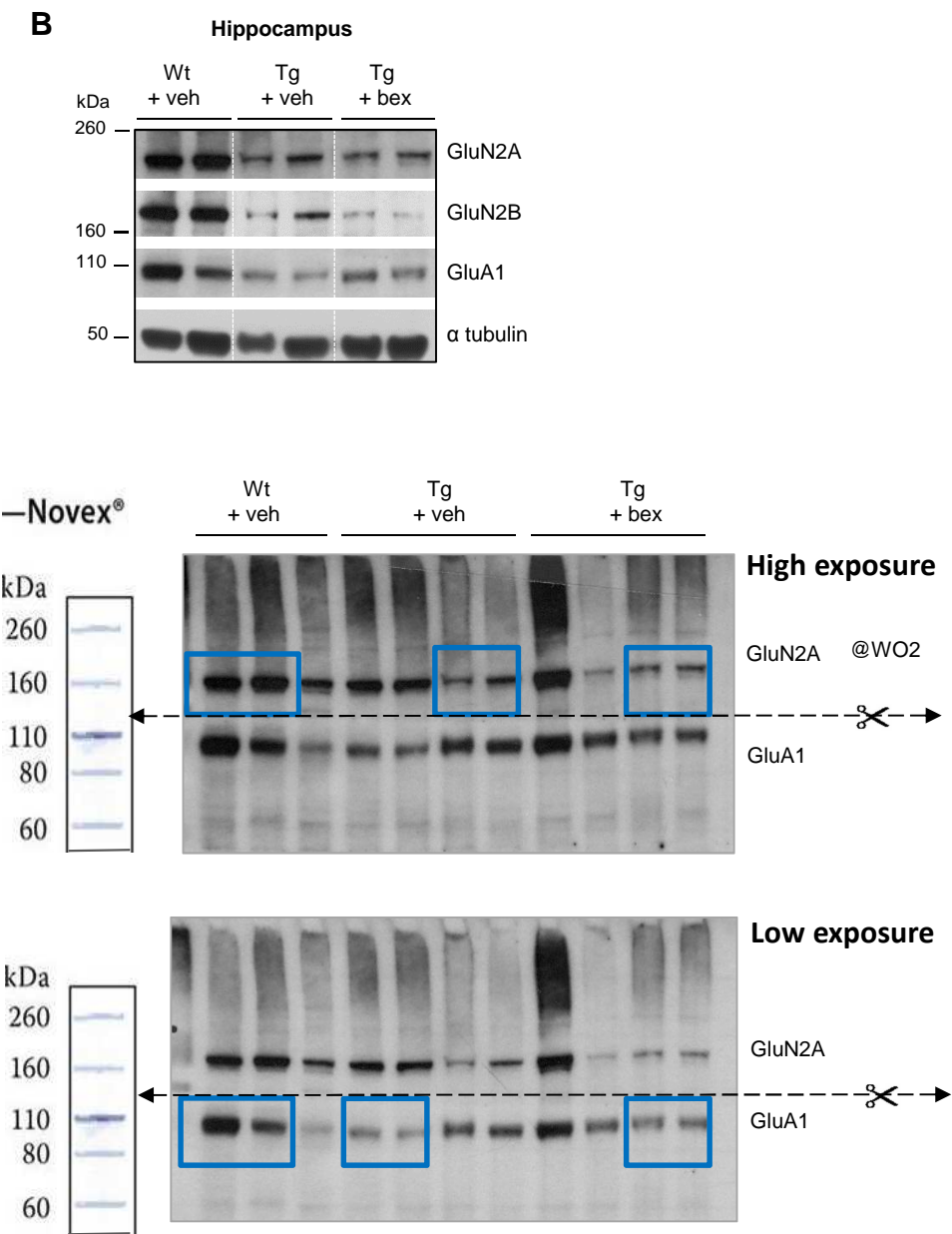

Figure 1

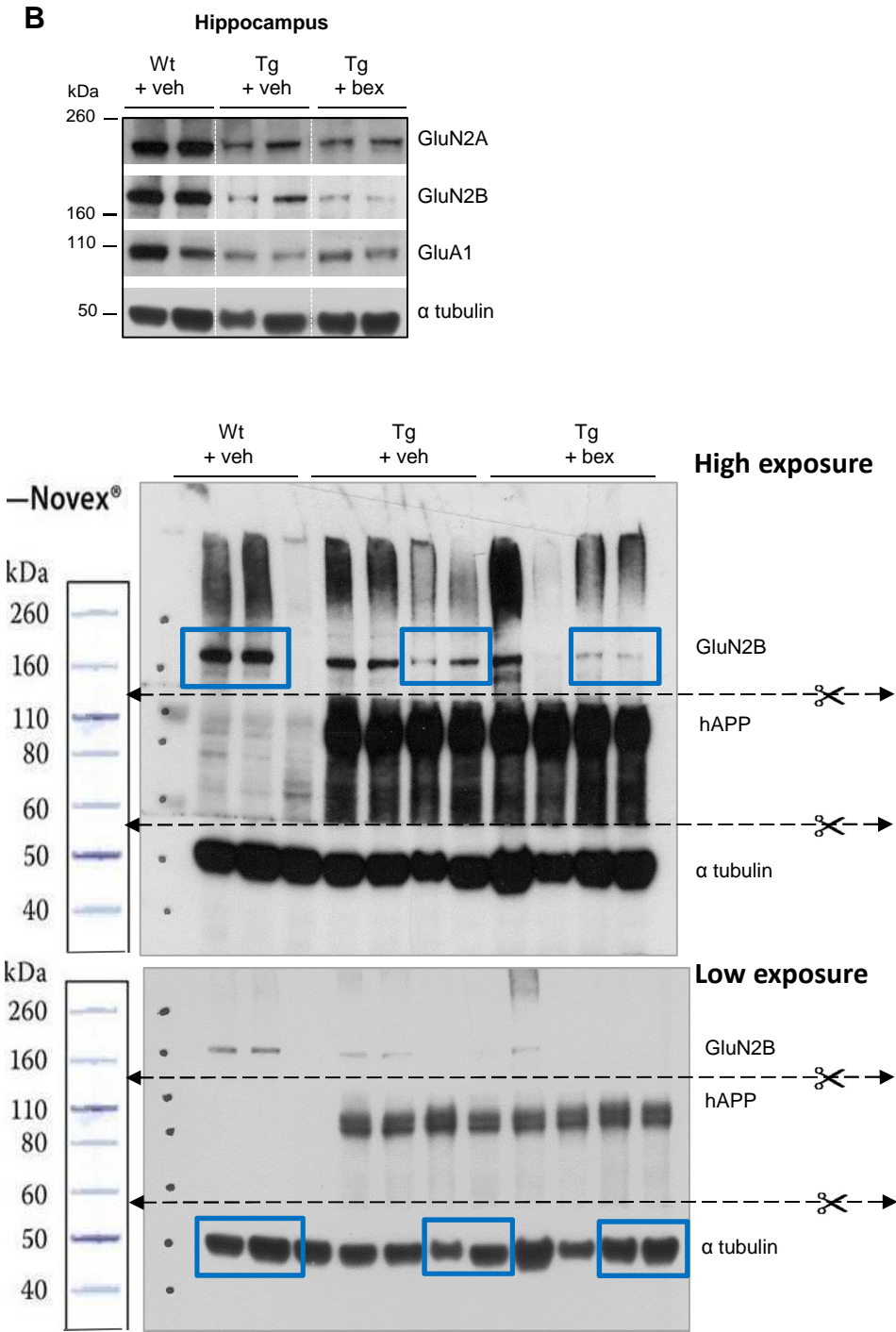

# **C cortical cells**

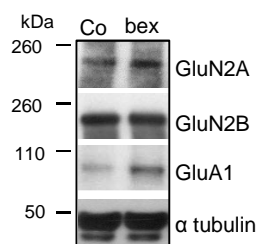

—Novex®

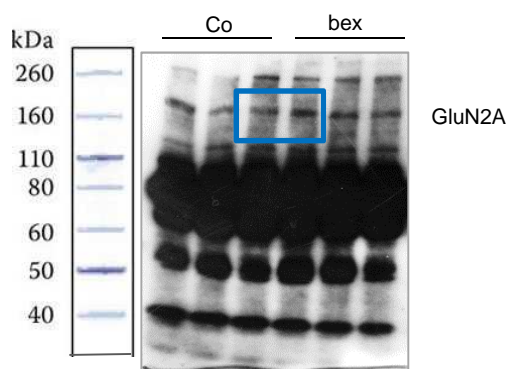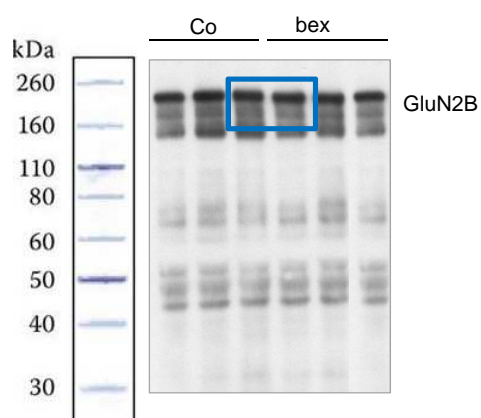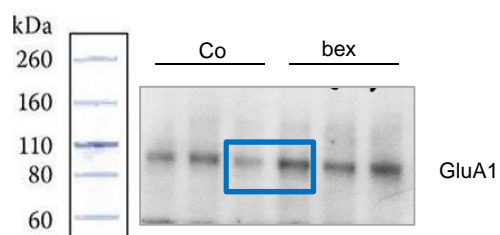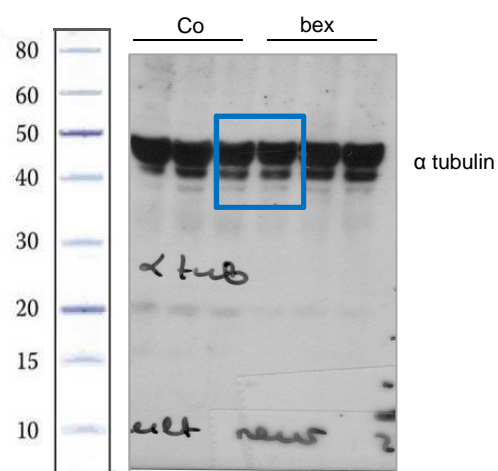

**Figure 1**

Figure 1

E

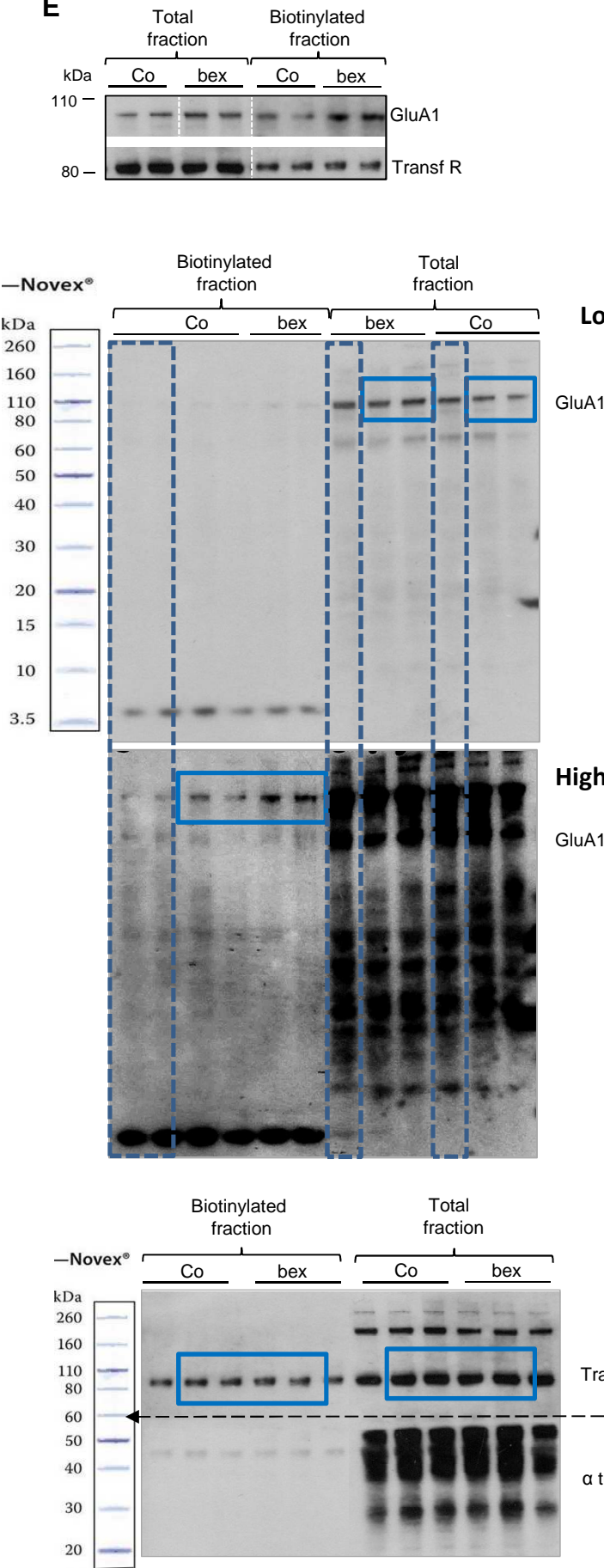

Supplement: Supplementary file 1 [file LSA-2018-00262_SdataF1.pdf]

Figure S2

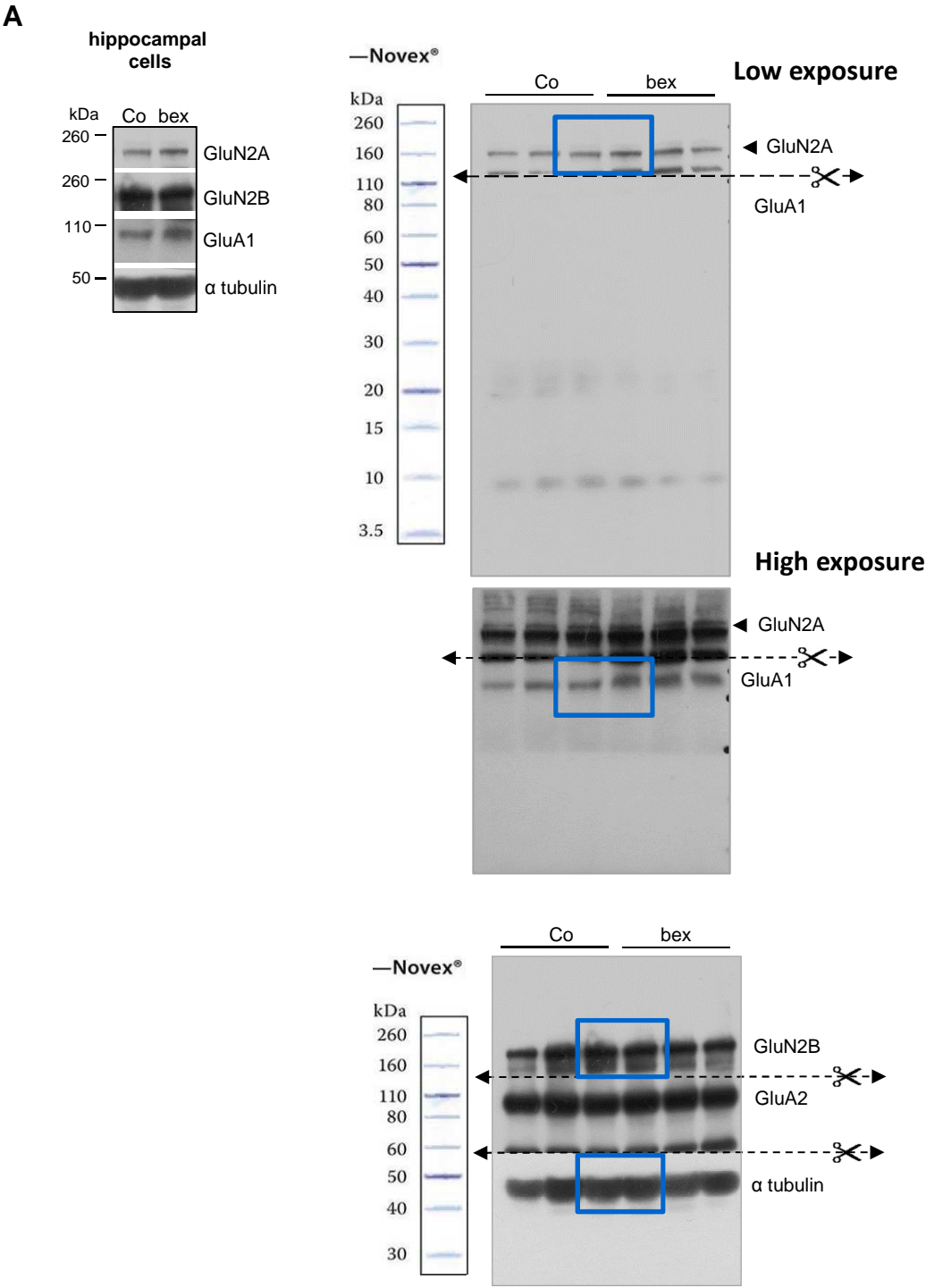

**B****Figure S2****hippocampal slices**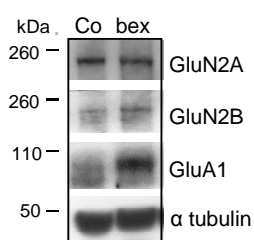**—Novex®**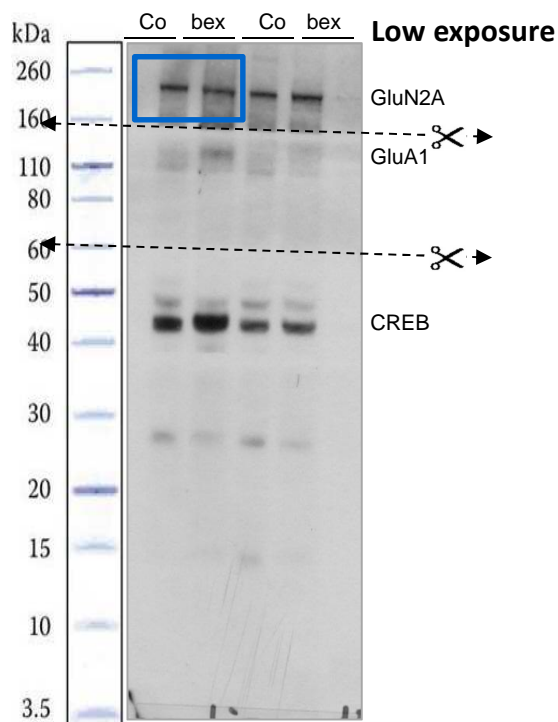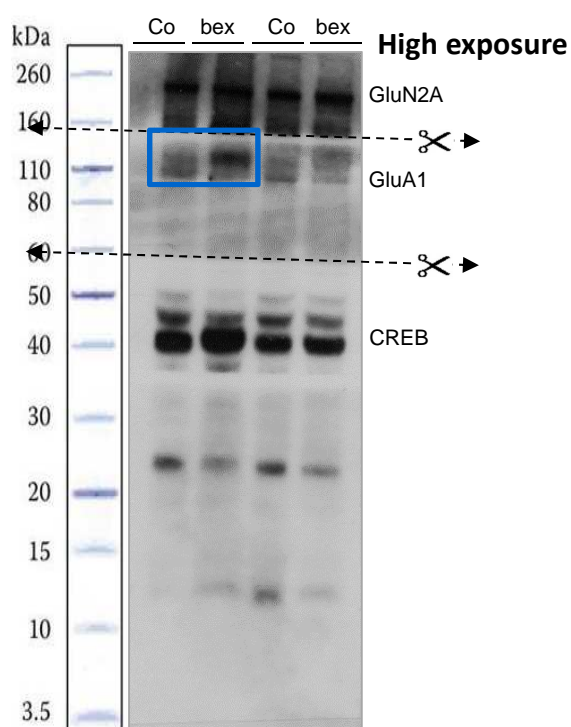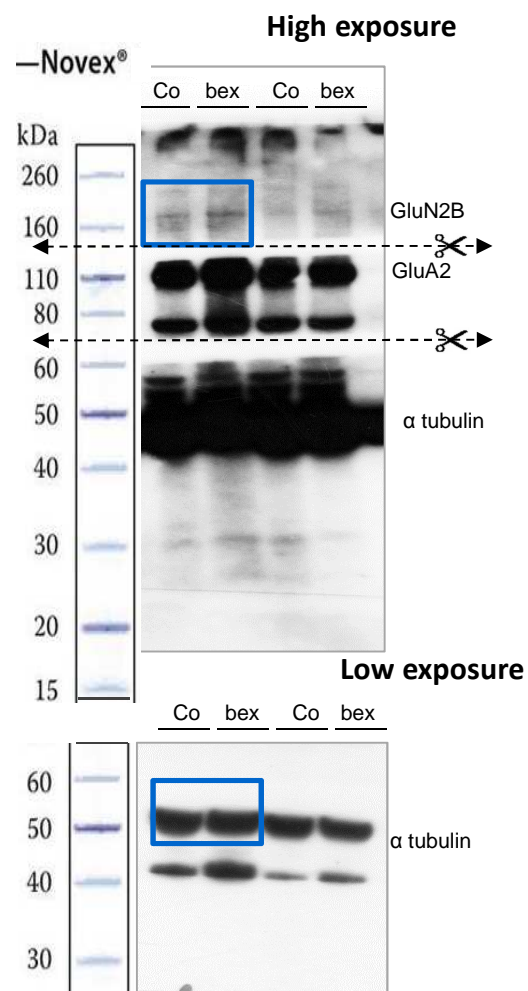

**C**

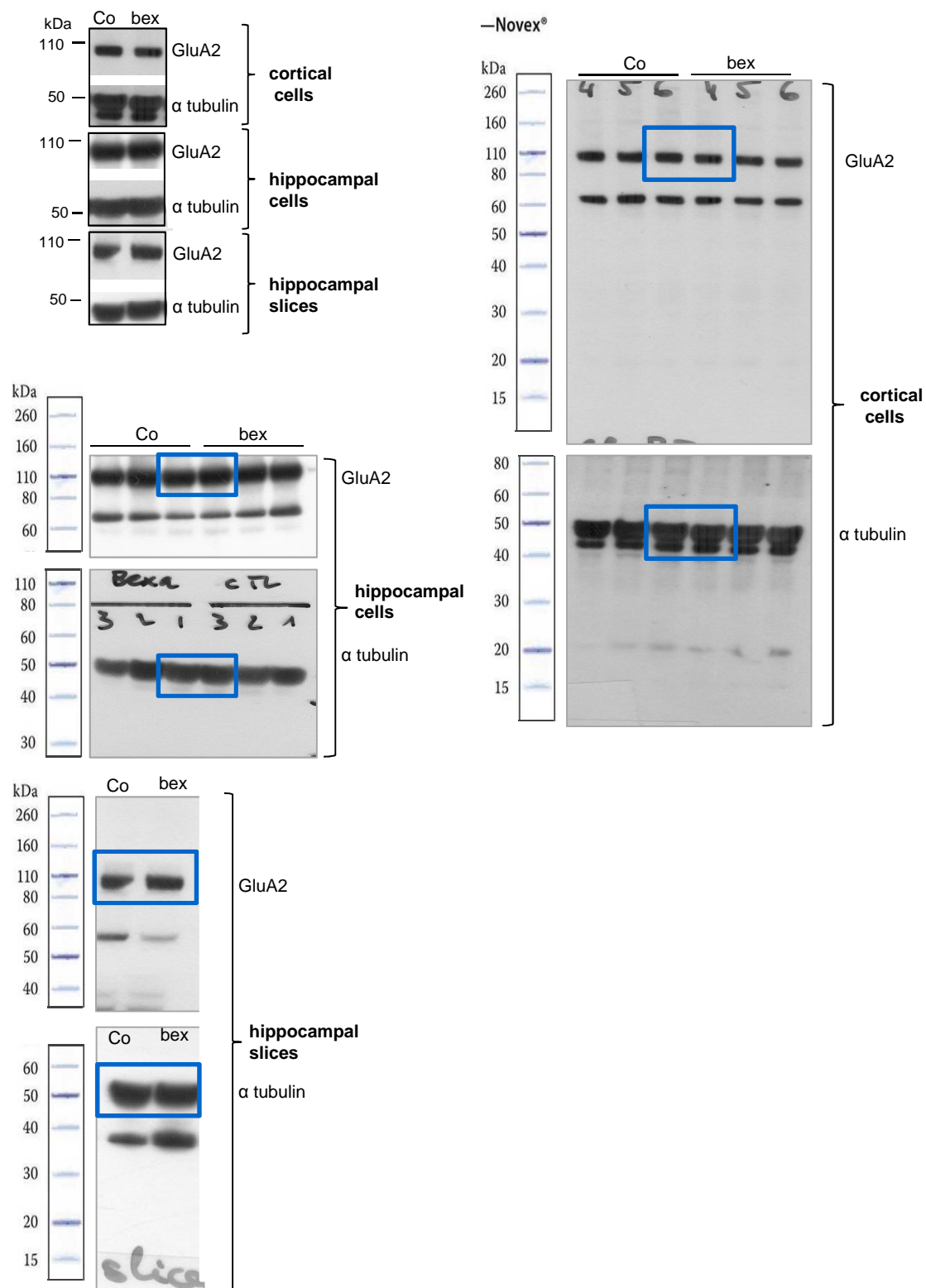

**Figure S2**

Supplement: Supplementary file 2 [file LSA-2018-00262_SdataFS2.pdf]

**C****Figure 2**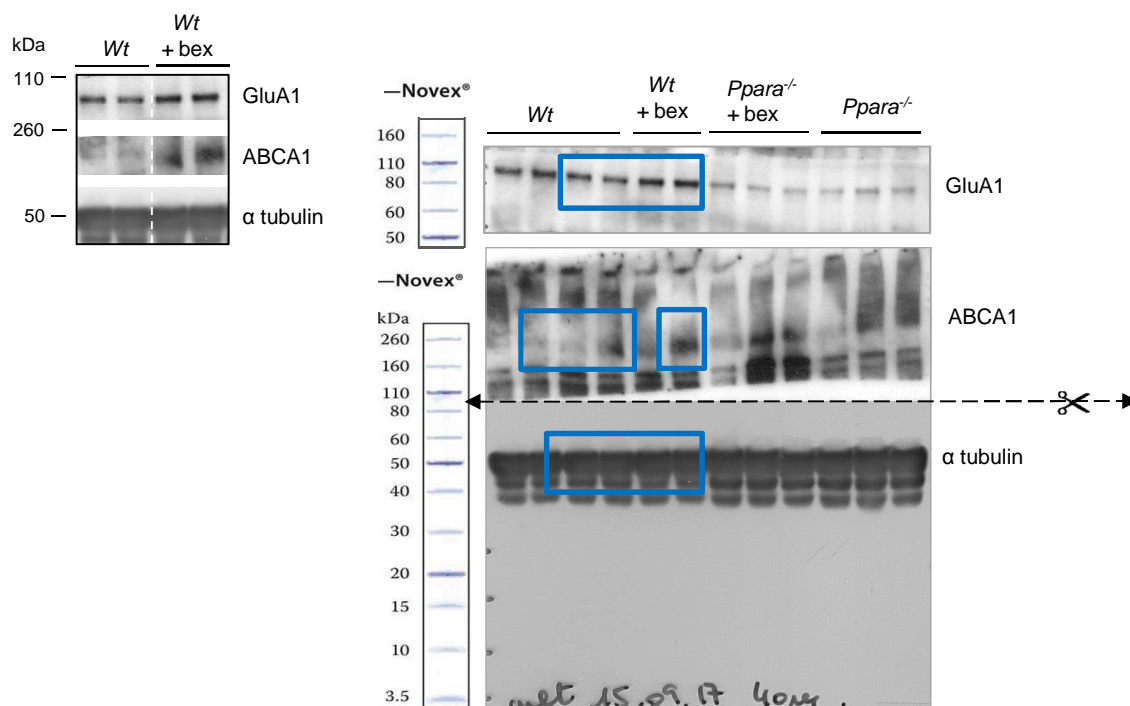

**D****Figure 2**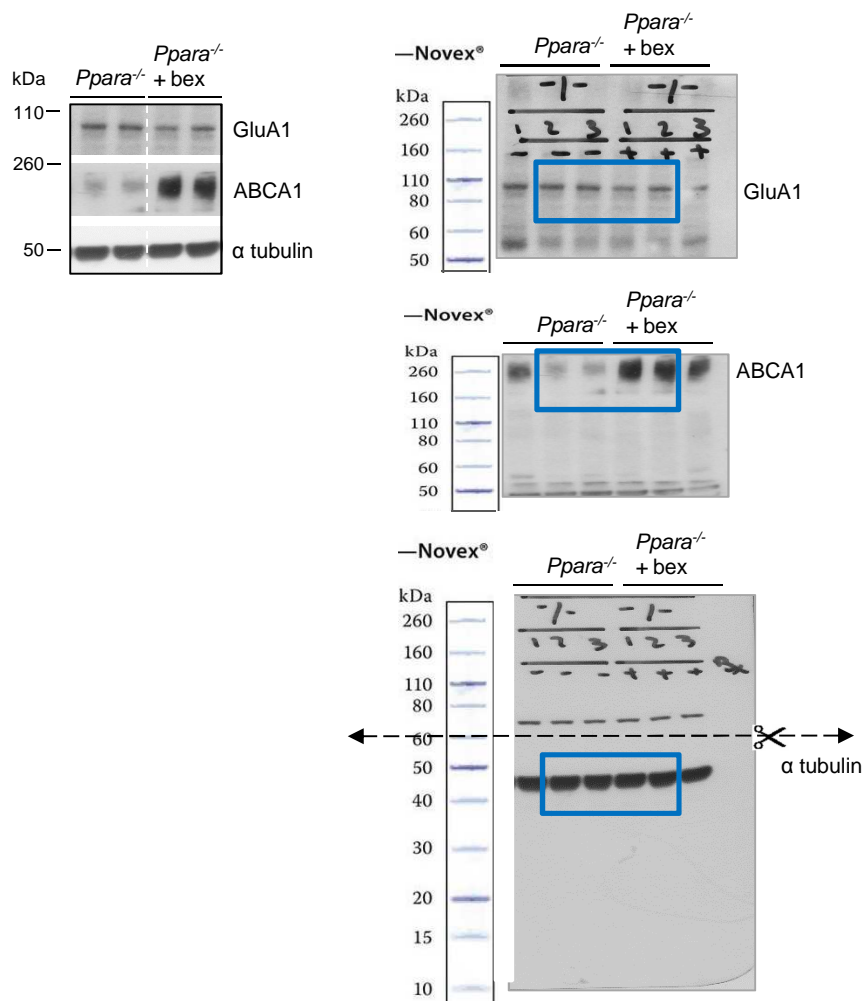

Supplement: Supplementary file 3 [file LSA-2018-00262_SdataF2.pdf]

**A**

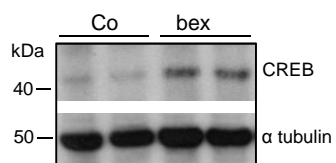

**Figure S3**

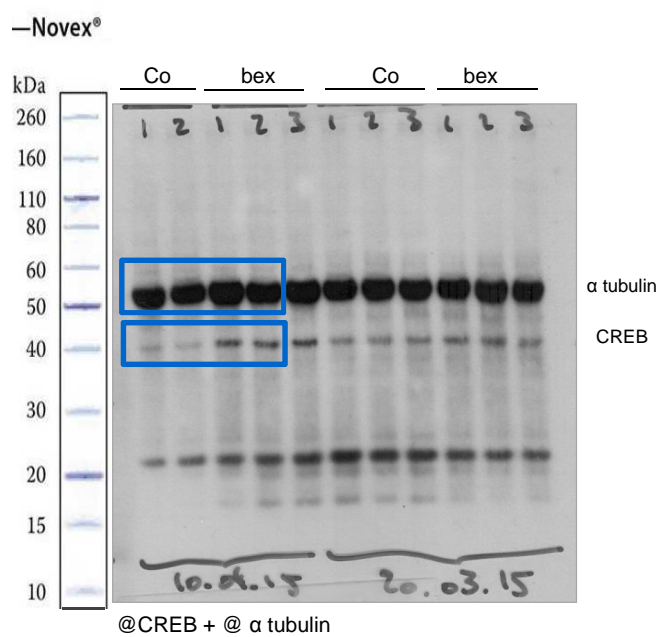

Supplement: Supplementary file 4 [file LSA-2018-00262_SdataFS3.pdf]

A

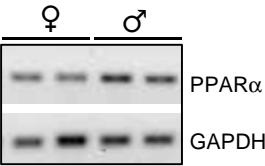

Figure 3

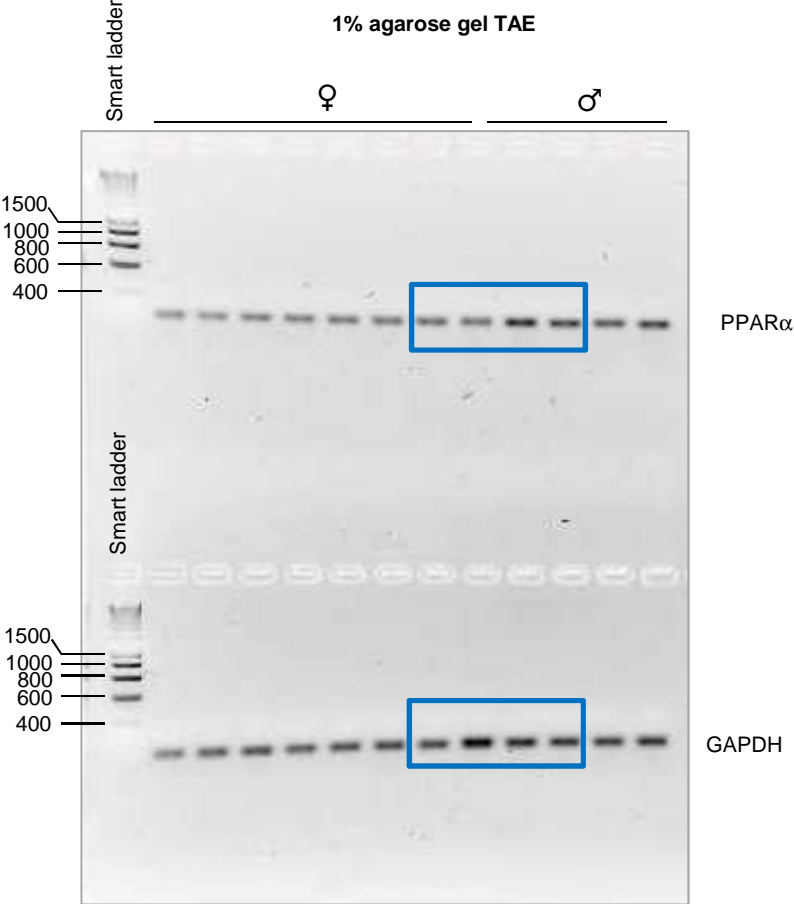

Figure 3

D

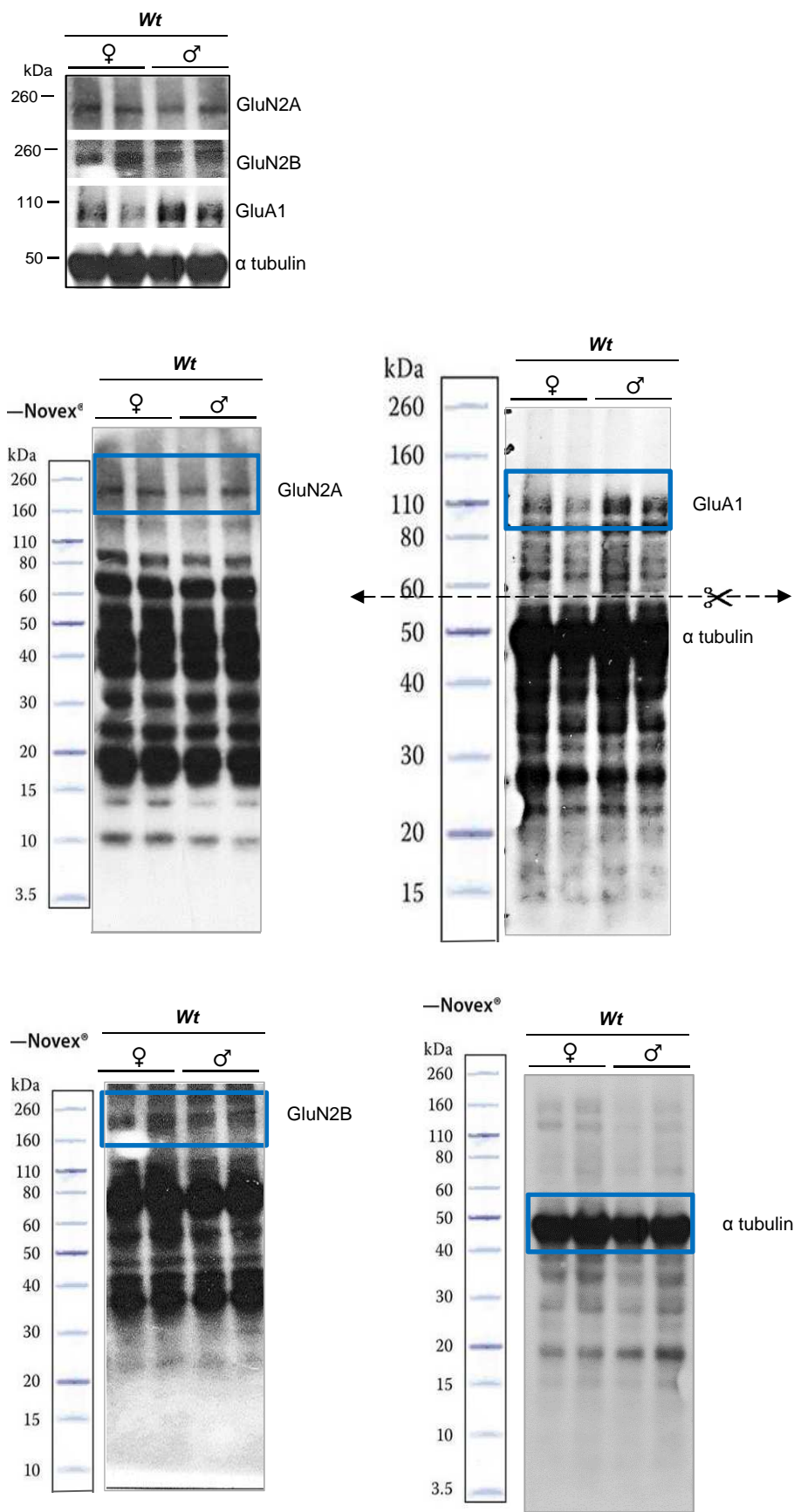

Figure 3

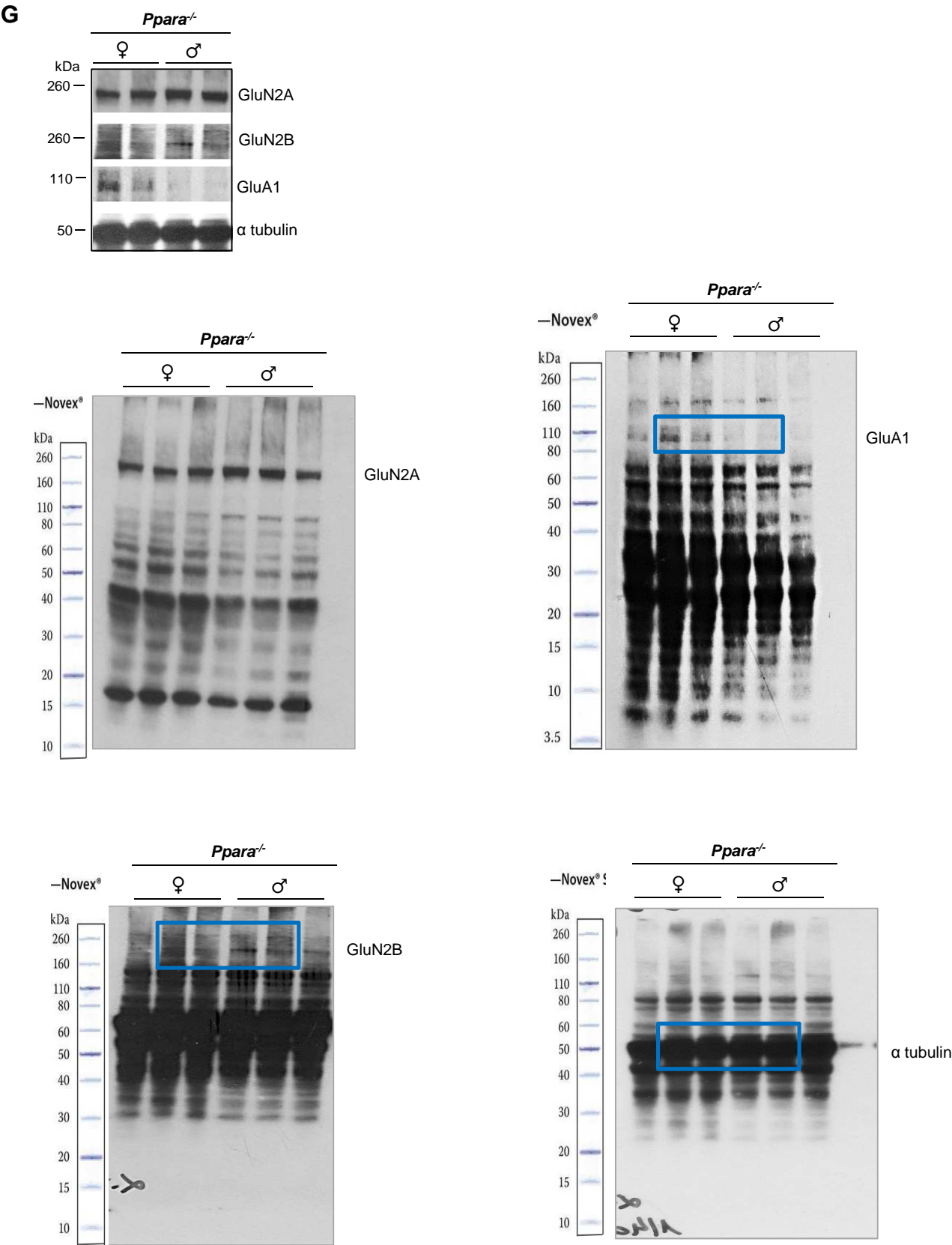

Supplement: Supplementary file 5 [file LSA-2018-00262_SdataF3.pdf]

**A**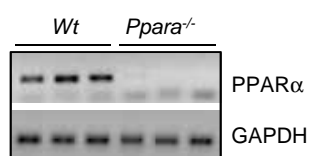**Figure S4**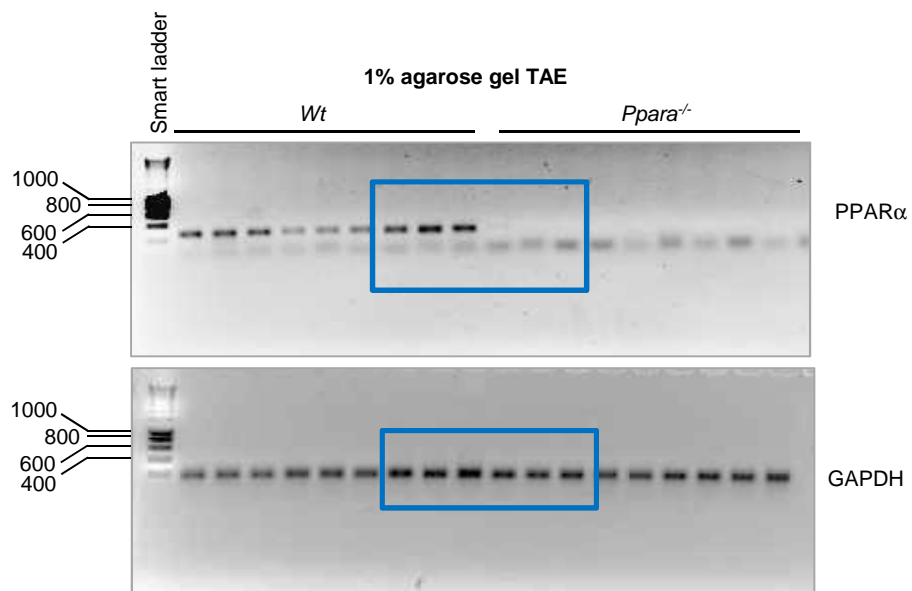

**D**

**Figure S4**

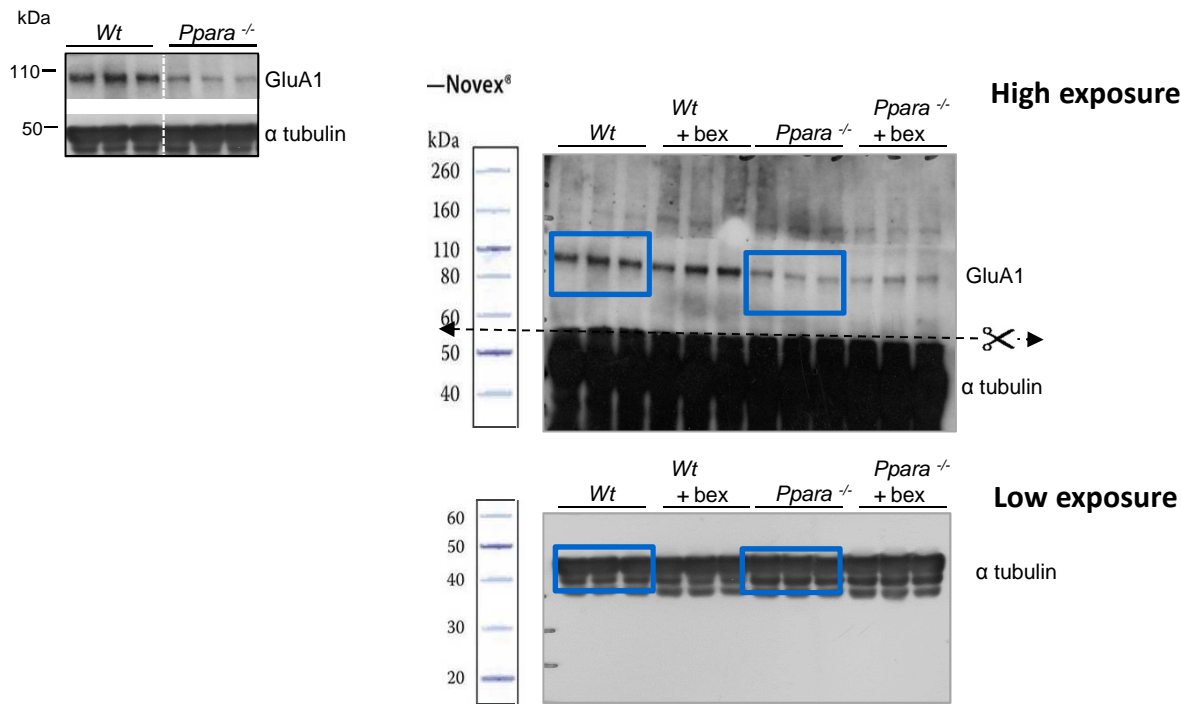

Supplement: Supplementary file 6 [file LSA-2018-00262_SdataFS4.pdf]

**Figure 4**

**B**

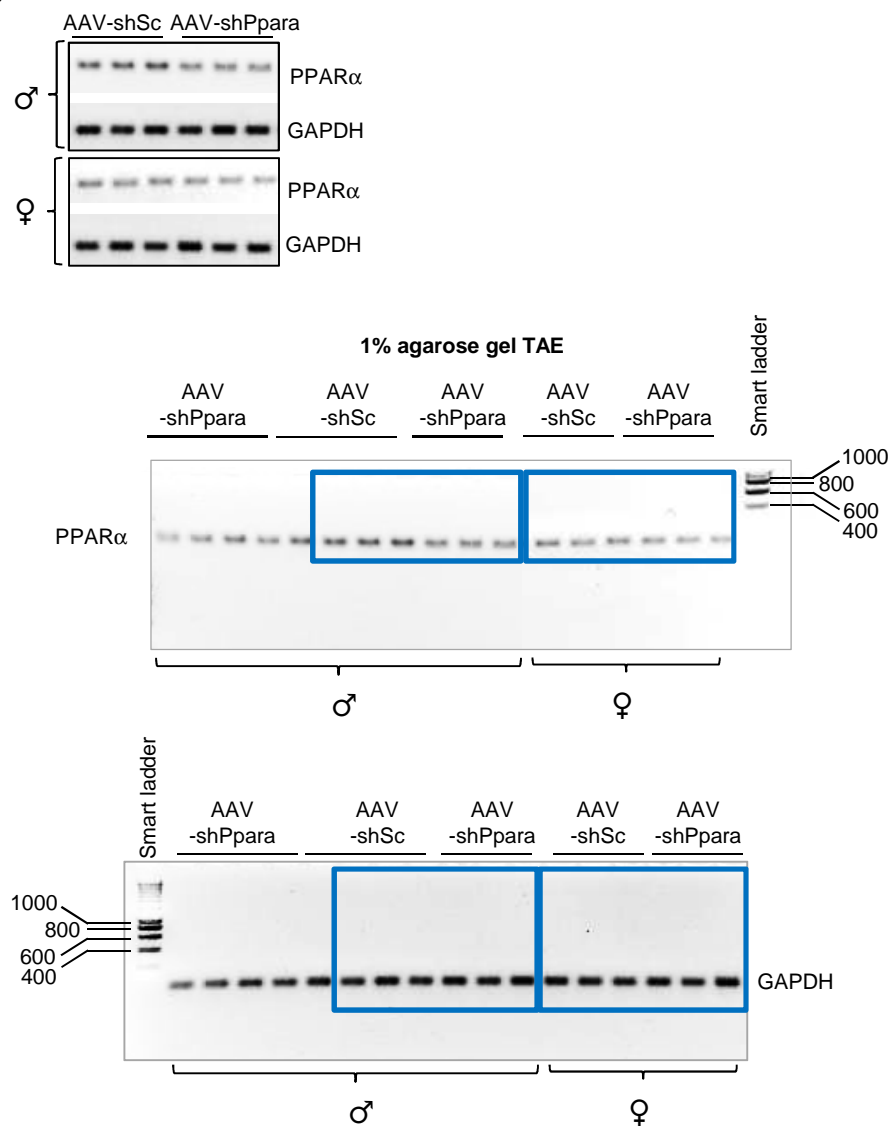

Figure 4

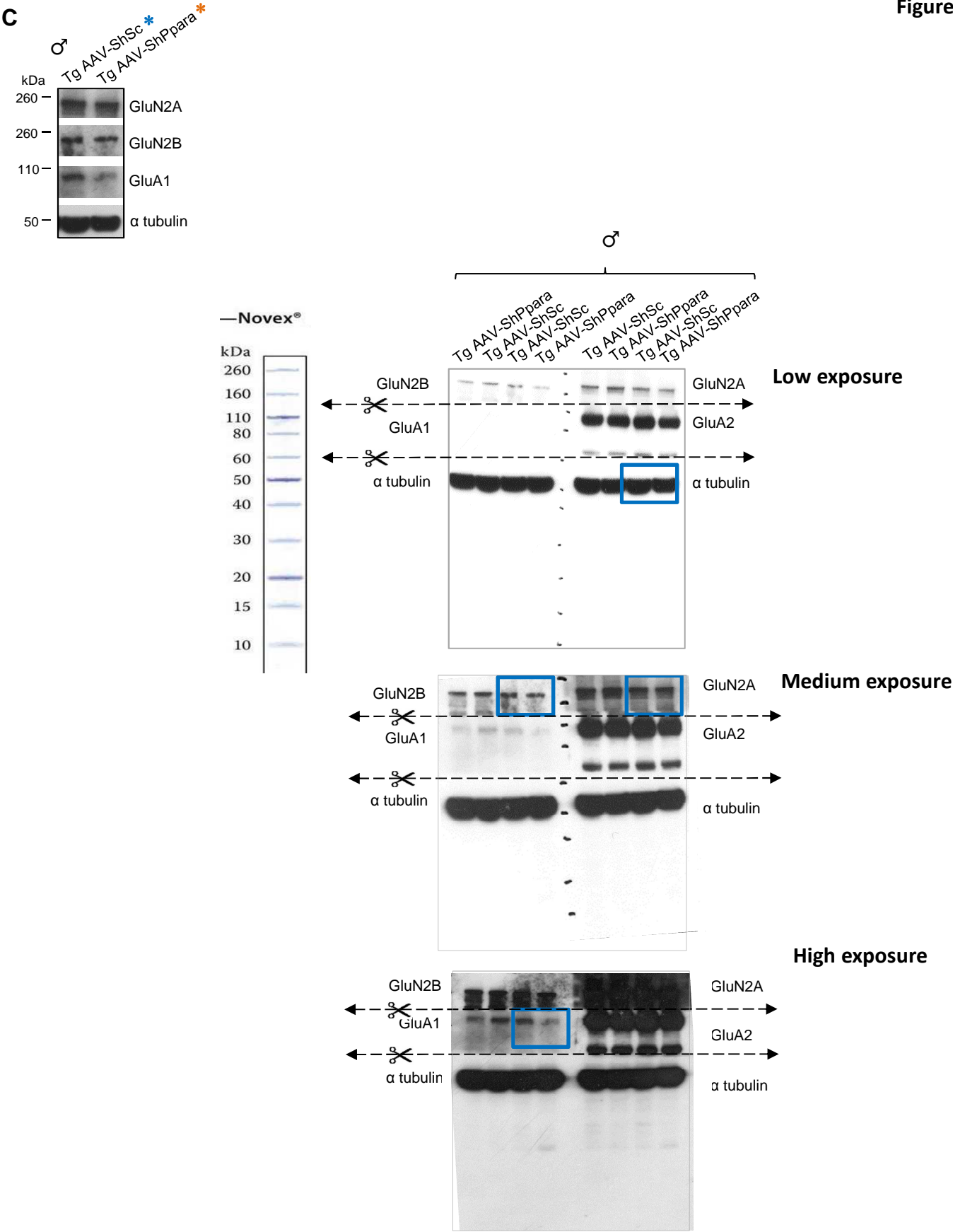

**D**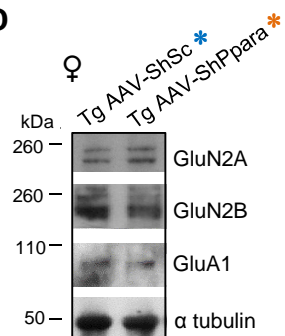**Figure 4**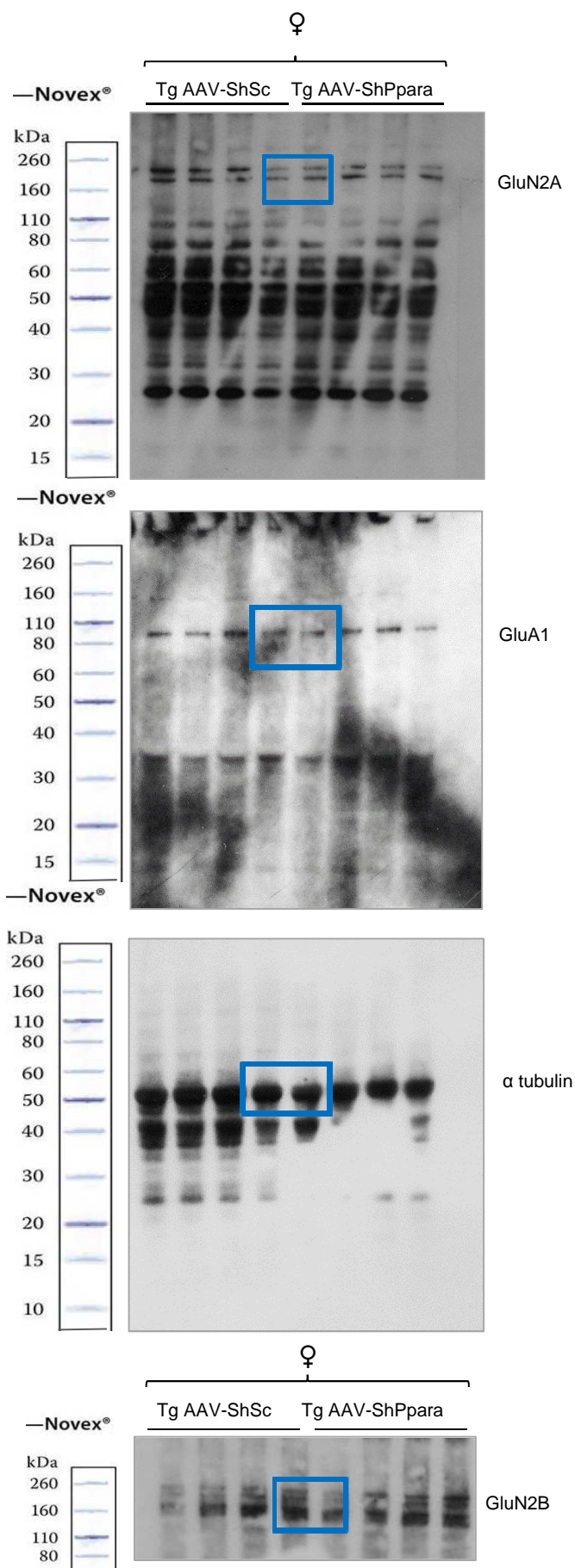

Supplement: Supplementary file 7 [file LSA-2018-00262_SdataF4.pdf]

Figure S5

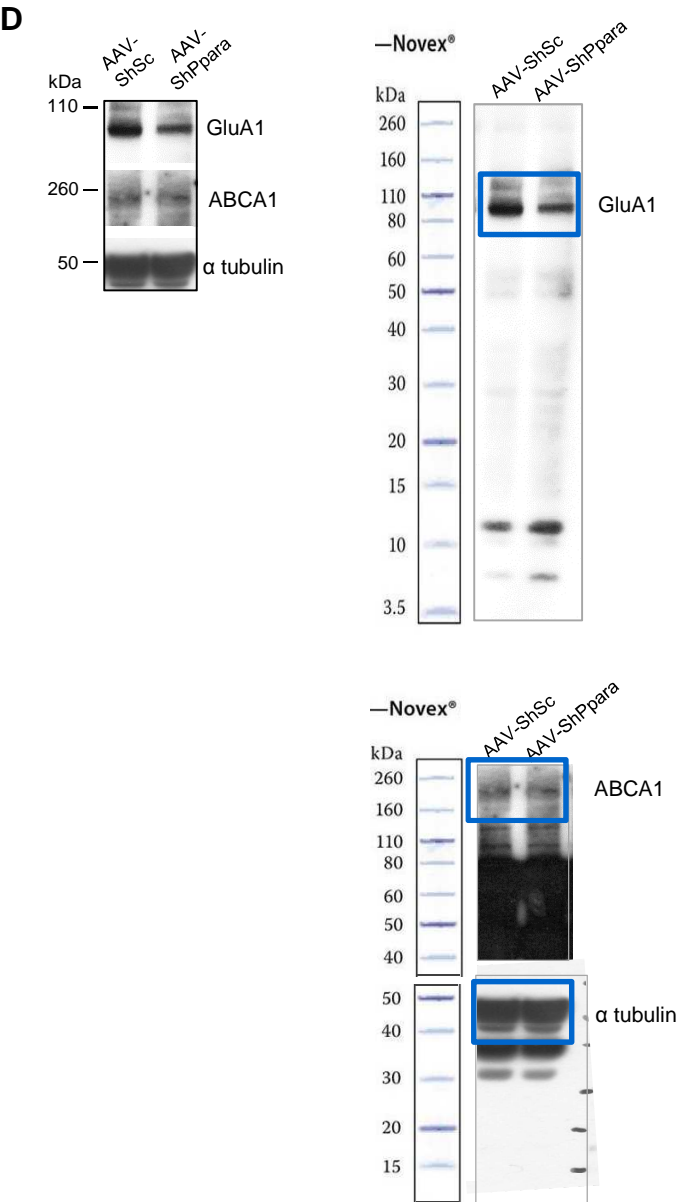

Supplement: Supplementary file 8 [file LSA-2018-00262_SdataFS5.pdf]

**Figure 5**

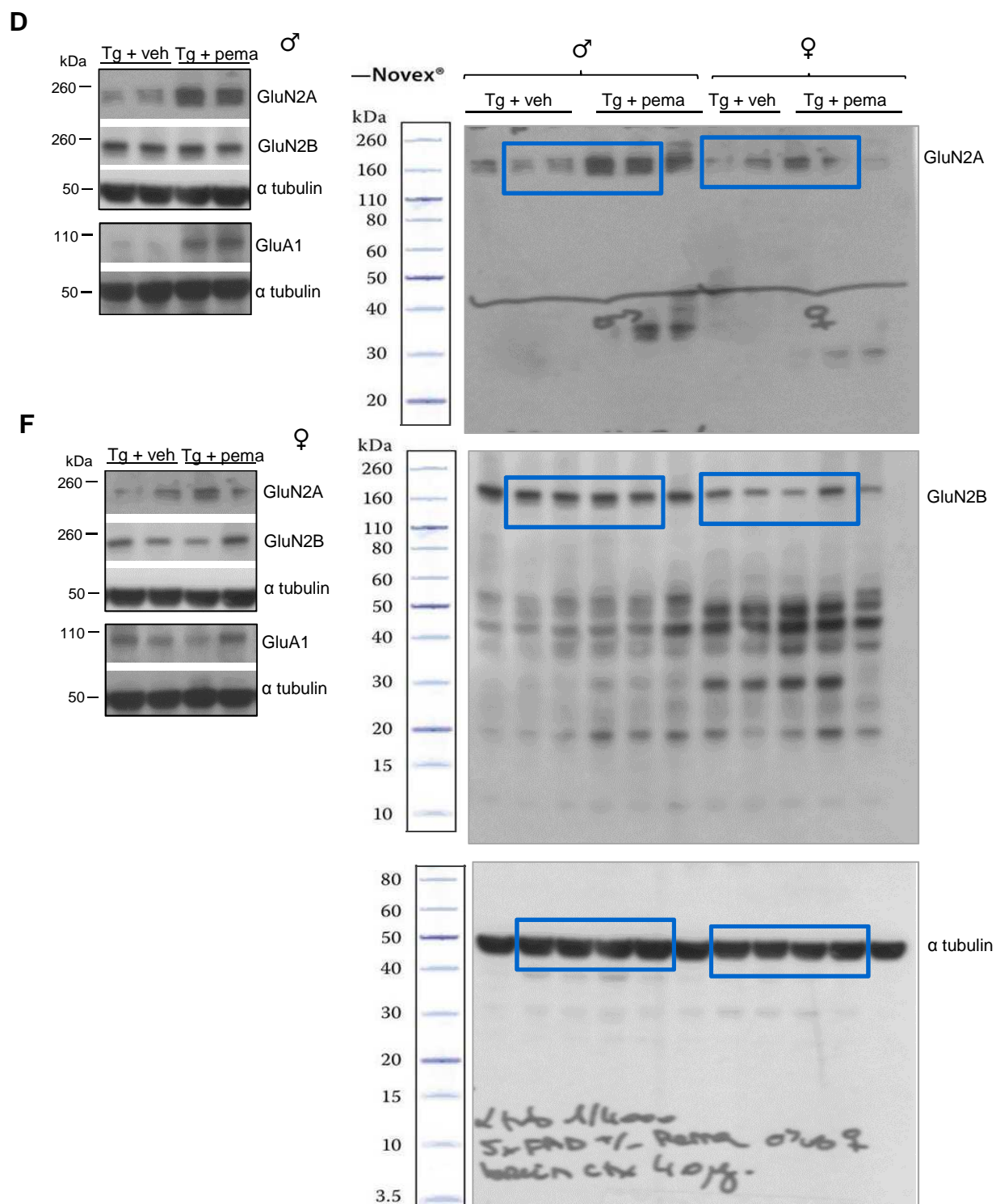

Figure 5

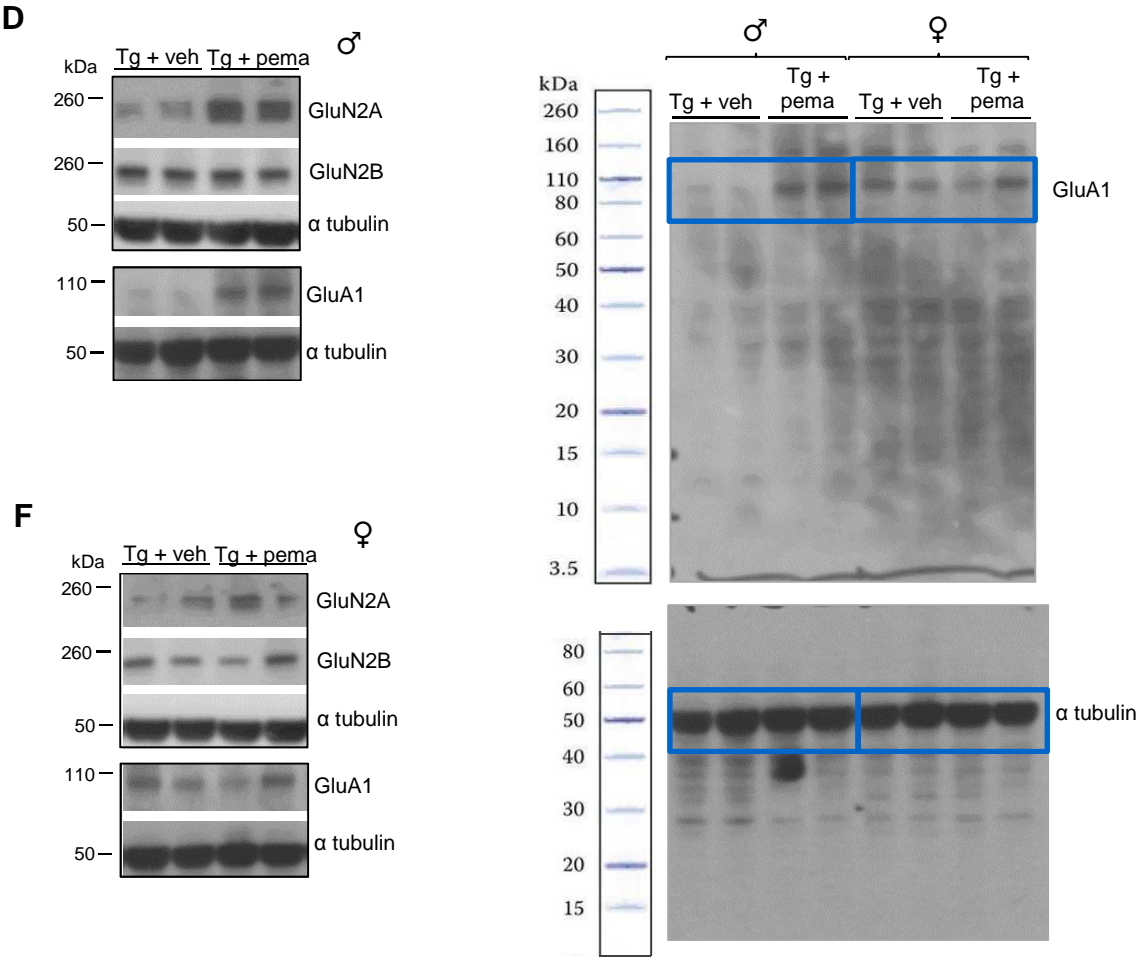

Supplement: Supplementary file 9 [file LSA-2018-00262_SdataF5.pdf]

Figure S6

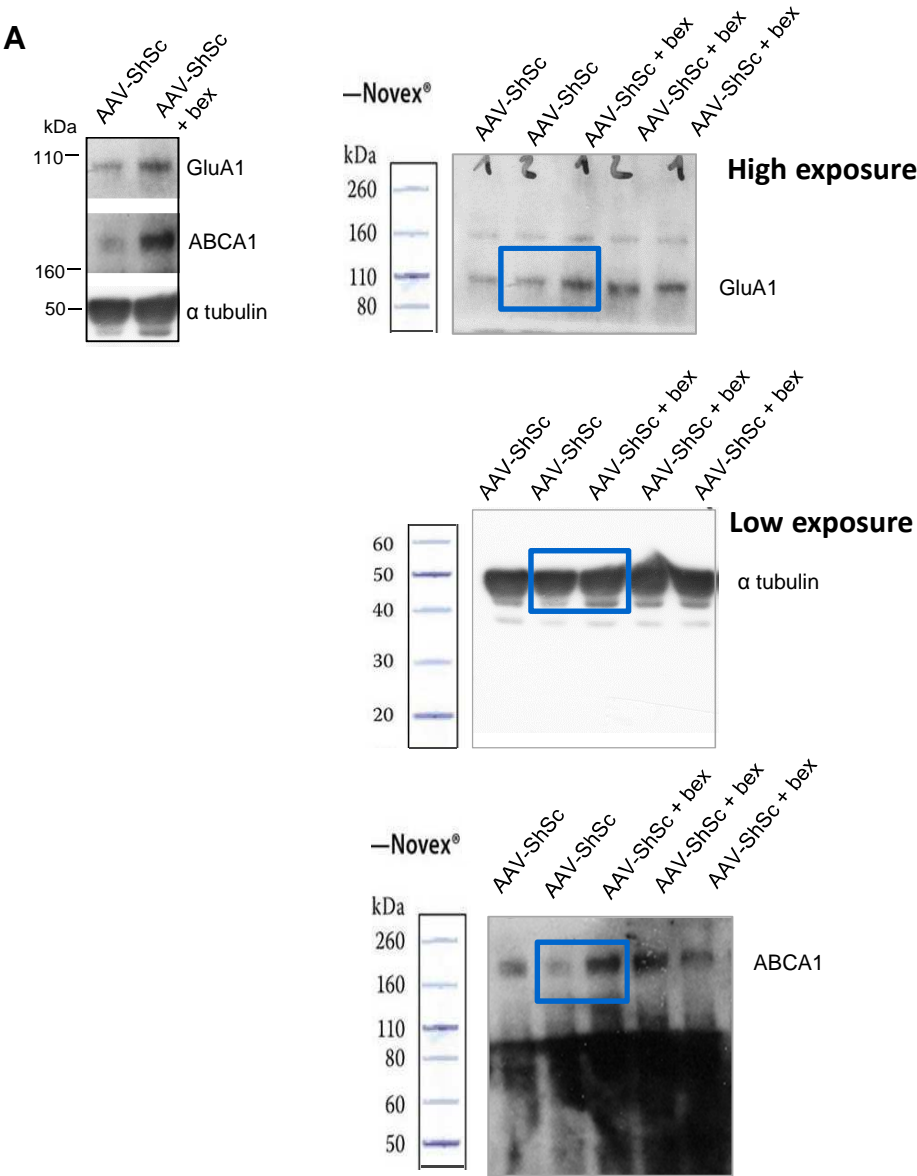

**B**

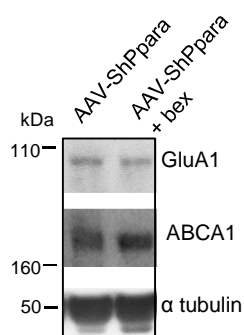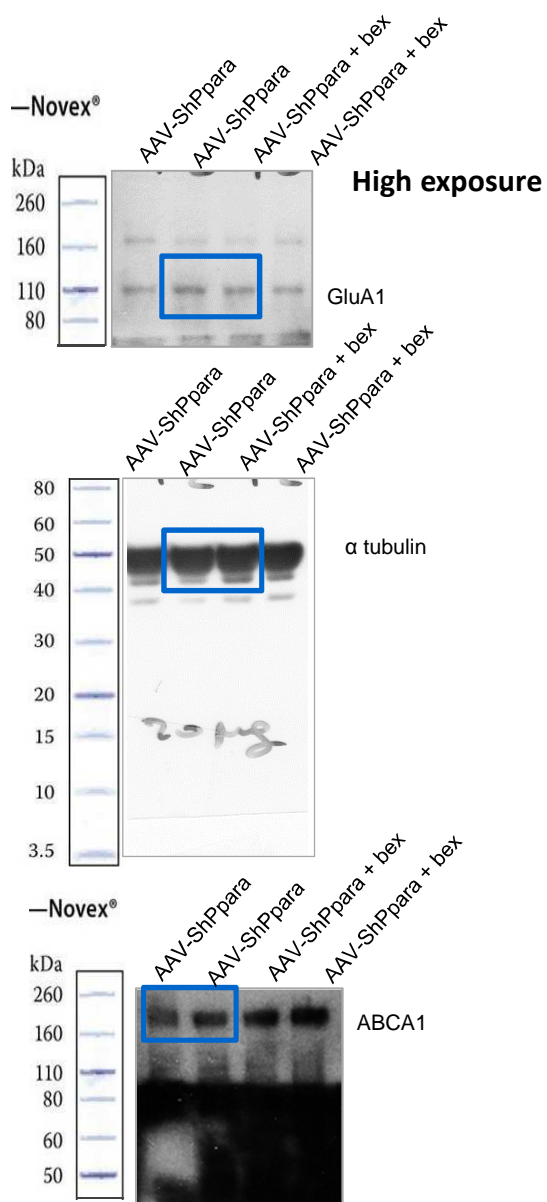

**Figure S6**

Supplement: Supplementary file 10 [file LSA-2018-00262_SdataFS6.pdf]

Figure S7

A

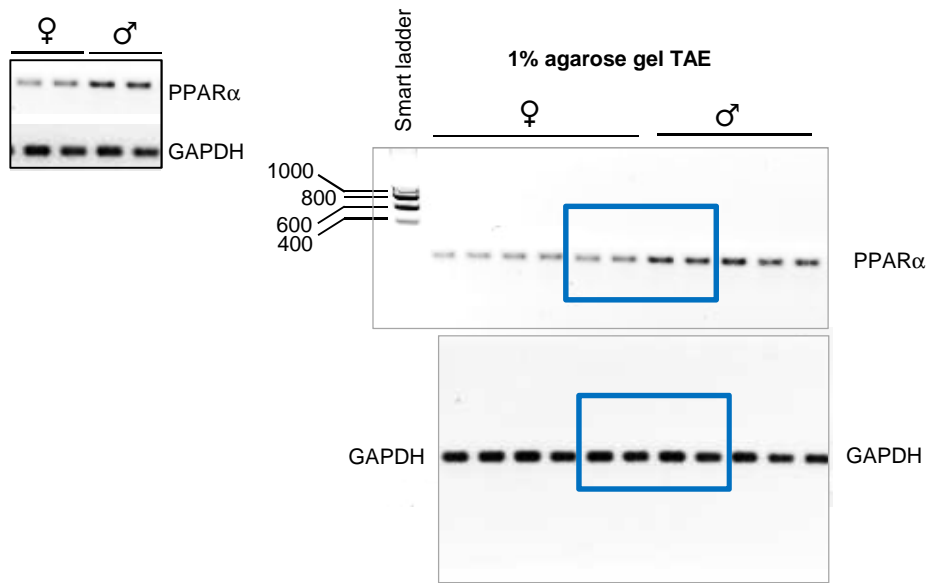

B

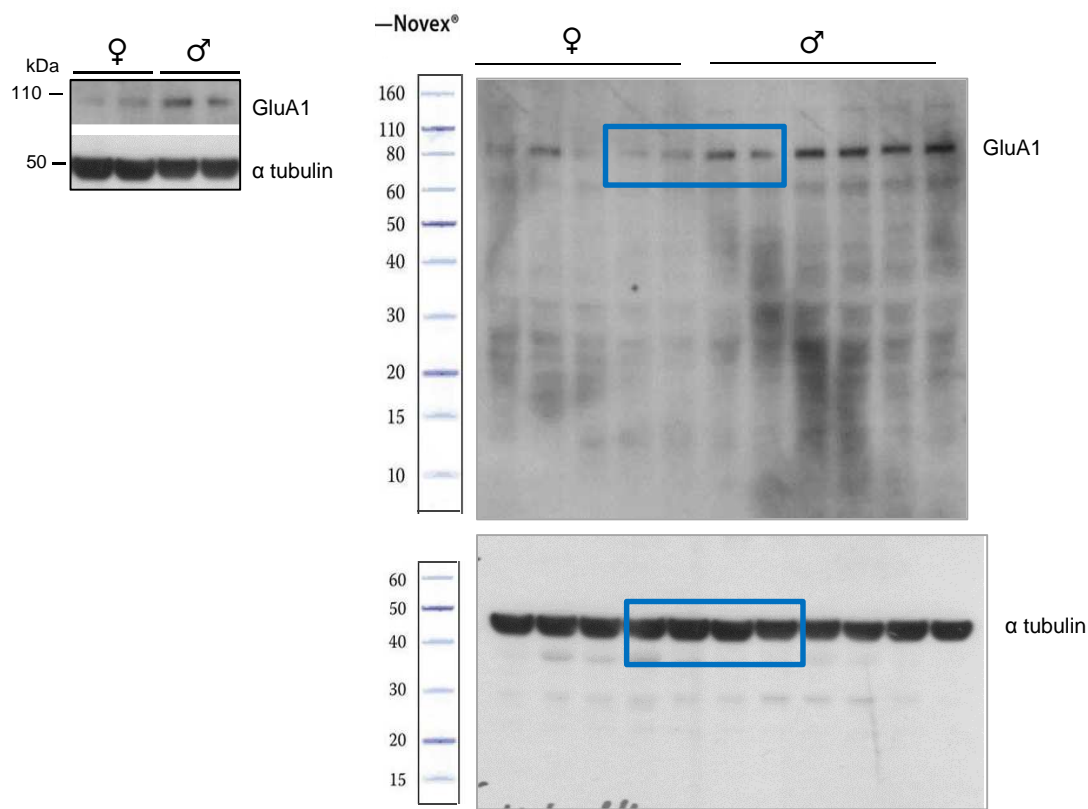

Supplement: Supplementary file 11 [file LSA-2018-00262_SdataFS7.pdf]
