# Supplementary material for: Sex-regulated gene dosage effect of PPARα on synaptic plasticity
Source: Life Sci Alliance. 2019 Mar 20;2(2):e201800262. doi: 10.26508/lsa.201800262 (PMC6427998; doi:10.26508/lsa.201800262)
Supplement: Supplementary file 12 [file LSA-2018-00262_TableS1.docx]

**Table S1. List of primers used for real-time PCR**
