# Supplementary material for: Sex-regulated gene dosage effect of PPARα on synaptic plasticity
Source: Life Sci Alliance. 2019 Mar 20;2(2):e201800262. doi: 10.26508/lsa.201800262 (PMC6427998; doi:10.26508/lsa.201800262)
Supplement: Supplementary file 13 [file LSA-2018-00262_Supplementary_Information.doc]

**Supplementary information**

**Supplementary Materials and Methods**

**Hippocampal organotypic tissue cultures**

Hippocampal organotypic slices were prepared from 7 to 8‐day‐old Wistar rat pups of internal breeding (Université catholique de Louvain, Brussels, Belgium) as previously described by (Paci et al, 2017; Stoppini et al, 1991). Briefly, rats were quickly decapitated and their brains were removed to isolate hippocampi in a cold sterile dissection medium (MEM with Earle's salts with L‐glutamine, 10 mM Trizma® base (Sigma-Aldrich, St-Louis, MO, USA, catalog no. 93362) and 1% penicillin‐streptomycin). The hippocampi were dissected and slices of 400 μm thickness were cut with a McIlwain Tissue Chopper (The Mickle Laboratory Engineering Co. LTD, UK). Selected slices were put on culture inserts (Merck Millipore, Burlington, MA, USA, catalog no. PICM0RG50) with culture medium (MEM with Earle's salts and L‐glutamine, 25% Horse serum, 25% HBSS with CaCl2 and MgCl2, 1% penicillin‐streptomycin, 5 mM Trizma® base, 0.41 mM NaHCO3, 1 mM sodium pyruvate, 10 mM HEPES). The slices were maintained in a humidified atmosphere at 37°C with 5% CO2 for 7 days. The culture medium was replaced every 2-3 days.

**Behavioral analysis**

Behavioural analysis was performed using standard protocols previously used to analyse AD mouse models (Dewachter et al, 2002; Postina et al, 2004). 5xFAD and age-matched controls mice were acclimatized to the behavioural facility prior to behavioural assessment. For the object recognition task (ORT), 5xFAD and age-matched controls mice at 9-10 months of age following vehicle (water, gavage, 10 days) or bexarotene treatment (100 mg/kg/day b.wt., gavage, 10 days) were submitted to a 10 min acquisition trial, following habituation to the open field box (10 min). During the acquisition trial, mice were placed individually in the open field in the presence of object A, and the time spent exploring object A (when the animal's snout was directed toward the object at a distance <1 cm) was measured. During a 10 min retention trial (second trial), which was performed 3h later, a novel object (object B) was placed together with the familiar object (object A) in the open field. The time (tA and tB) the animal spent exploring the two objects was recorded. The recognition index (RI), defined as the ratio of the time spent exploring the novel object over the time spent exploring both objects [(tB/(tA+ tB)) × 100] was used to measure object recognition. For the spatial navigation task, all animals were habituated to the behavioural facility for at least one week before performing the experiments.

A modified version of the Morris water maze with a mild learning paradigm was used. The Morris water maze test was essentially performed as previously described (Stancu et al, 2014). A circular polypropylene pool (113 cm in diameter), filled with opaque water (26 ± 1°C) for hiding the submerged escape platform (9.5 cm diameter) was used for testing. For place navigation tests, mice were trained to locate the hidden platform in three trials per day, each with duration of maximum 60s, with an inter-trial interval of 1 hour. Training over five consecutive days was performed. Spatial navigation analysis started following 5 days of treatment with vehicle (water, gavage, 10 days) or bexarotene (100 mg/kg/day b.wt., gavage, 10 days), until day 10. Before the spatial navigation test, the mice were submitted to a cued navigation task, in which the mice were trained to locate a visible platform within a period of 60s. Mouse behavior in the pool was recorded and tracked using Ethovision camera and software (EthoVision 6.1 Noldus, Wageningen, The Netherlands). After behavioral experiments, mice were sacrified and LTP was measured.

**RNA extraction and real time PCR**

Real-time PCR was performed for the amplification of cDNAs with specific primers (F, Forward and R, Reverse, Sigma-Aldrich (St-Louis, MO)) for:

Creb1 mouse (RefSeq: NM_133828) and rat (RefSeq: NM_031017): mouse/rat F-5’GGAGCAGACAACCAGCA3’, mouse/rat R-5’ACTGTTTGGACTT GTGGAGAC3’; Pdk4 mouse (RefSeq: NM_013743): F-5’ACACGCTGGTCAAAGTTC3’, R-5’ TGAGCATCCGAGTAGAAAT3’. Real-time PCR was carried out in a total volume of 25 μL containing 16 ng cDNA template, 0.3 μM of the appropriate primers and the IQTM SYBR® Green Supermix 1x (Bio-Rad Laboratories, Hercules, CA, USA, catalog no. 1708885).

**Semi-quantitative RT-PCR**

Total RNA was isolated from primary cultures of mouse cortical cells prepared from wild type and Ppara deficient mice using TriPure Isolation Reagent (Roche, Basel, Switzerland, catalog no. 11667165001) following manufacturer's protocol. Briefly, 1 μg of total RNA was reverse-transcribed (see RNA extraction section). Semi-quantitative RT-PCR was carried out after treating total RNA with DNAse to remove any contaminating genomic DNA. The resulting cDNA was appropriately diluted and amplified using TaqDNA polymerase and the following primers (F, Forward and R, Reverse, Sigma-Aldrich (St-Louis, MO)): Ppara mouse (Refseq: NM_011144): mouse F-5’AAACTTGGACTTGAACGACC3’, mouse R-5’GCATCCCGTCTTTGTTCA3’; Gapdh mouse (RefSeq: NM_008084): F-5’CATGGCCTTCCGTGTTCCTA3’, R-5’GCGGCACGTCAGATCCA3’. Amplified products were electrophoresed on a 2% agarose gel and visualized by Midori Green Advance DNA staining (Nippon Genetics Europe, Eupen, Belgium, catalog no. MG04) with an electrophoresis gel imaging system (Bio-Rad GelDoc 2000, Bio-Rad Laboratories, Hercules, CA, USA).

**Western blotting**

Cell lysates (40 µg of proteins) were analyzed by Western blotting using 4-12 % NupageTM bis-Tris gels. Nitrocellulose membranes were incubated overnight at 4 °C with the following primary antibody: mouse monoclonal anti-CREB (1:1000) (86B10, Cell Signaling Danvers, MA, USA, catalog no. 9104).

**Immunocytochemistry**

Cells were seeded at 105 cells/cm2 on 15 mm round glass coverslips pre-coated with 10 µg/ml poly-L-lysine in PBS, fixed 15 min with 4% v/v formaldehyde at room temperature then washed in PBS and permeabilized 1 h with 0,4% Triton X100 (v/v) in PBS containing 3% bovine serum albumin (Sigma-Aldrich, St-Louis, MO, USA, catalog no. A7906). After three washes in PBS, cells were incubated 1h at room temperature with primary antibodies: rabbit polyclonal anti-PPAR alpha antibody (Rockland Immunochemicals Inc., Limerick, PA, USA catalog no. 600-401-421) (anti-PPARα 1:200), anti-MAP2 (1:1000), rabbit polyclonal anti-GFAP (Santa Clara, CA, USA, catalog no. Z0334) (1:1000) and anti-CREB (1:200). After three PBS washes, cells were incubated for 1h with 5 µg/ml Alexa-labelled secondary antibodies (1:200). After three additional PBS washes, preparations were mounted in EverBrite™ (VWR, Oud-Heverlee, Belgium, catalog no. 23003). Pictures were acquired with an AMG Evos fluorescence digital inverted microscope (Advanced Microscopy Group, Mill Creek, WA, USA).

**Immunohistochemistry**

Male 5xFAD mice at 9 months of age were anesthetized with pentobarbital (Nembutal, i.p. 100 mg/kg b.wt.) and brains were removed rapidly and fixed in 4% paraformaldehyde solution before subsequent immunohistochemical analysis. Free-floating sagittal sections (50 µm) were cut on a vibrating HM650V microtome (ThermoScientific). After three PBS washes, slices were fixed in PBS:methanol (v/v) 10 min with mild shaking at room temperature. Slices were then permeabilized with 0.1% Triton X100 (v/v) in PBS containing 3% bovine serum albumin for 1h. Immunohistochemical staining was performed on free-floating section according to standard protocols (Dewachter et al, 2002; Jawhar et al, 2010) using anti-Aβ (WO2, 1:1000, Merck Millipore, Burlington, MA, USA, catalog no MABN10) and appropriate Alexa-labelled secondary antibodies (1:200). Preparations were mounted in Fluoprep (Biomérieux, Marcy l' Etoile, FR, catalog no. 75521). Amyloid plaque load was analysed as described previously (Dewachter et al, 2002; Postina et al, 2004). Well-defined sagittal sections at 1.44 mm lateral from bregma were selected for quantification of plaques. Plaques were quantified by measuring the area occupied by amyloid plaques relative to total brain area in hippocampus, CA1, cortex and subiculum, using Image J software on 10x digital images obtained with an AMG Evos fluorescence digital inverted microscope (Advanced Microscopy Group, Mill Creek, WA, USA).

**Supplementary references**

Dewachter I, Reverse D, Caluwaerts N, Ris L, Kuiperi C, Van den Haute C, Spittaels K, Umans L, Serneels L, Thiry E et al (2002) Neuronal deficiency of presenilin 1 inhibits amyloid plaque formation and corrects hippocampal long-term potentiation but not a cognitive defect of amyloid precursor protein [V717I] transgenic mice. J Neurosci 22: 3445-3453

Jawhar S, Trawicka A, Jenneckens C, Bayer TA, Wirths O (2010) Motor deficits, neuron loss, and reduced anxiety coinciding with axonal degeneration and intraneuronal Abeta aggregation in the 5XFAD mouse model of Alzheimer's disease. Neurobiol Aging

Paci P, Gabriele S, Ris L (2017) A new method allowing long-term potentiation recordings in hippocampal organotypic slices. Brain Behav 7: e00692

Postina R, Schroeder A, Dewachter I, Bohl J, Schmitt U, Kojro E, Prinzen C, Endres K, Hiemke C, Blessing M et al (2004) A disintegrin-metalloproteinase prevents amyloid plaque formation and hippocampal defects in an Alzheimer disease mouse model. J Clin Invest 113: 1456-1464

Stancu IC, Ris L, Vasconcelos B, Marinangeli C, Goeminne L, Laporte V, Haylani LE, Couturier J, Schakman O, Gailly P et al (2014) Tauopathy contributes to synaptic and cognitive deficits in a murine model for Alzheimer's disease. FASEB J 28: 2620-2631

Stoppini L, Buchs PA, Muller D (1991) A simple method for organotypic cultures of nervous tissue. J Neurosci Methods 37: 173-182
